# Simultaneous Assessment of Skeletal Muscle Energetics and Blood Flow During Dynamic Exercise by Interleaved ^31^P‐MRS/ ^1^H‐MRI

**DOI:** 10.1002/mrm.70337

**Published:** 2026-03-17

**Authors:** T. Jake Samuel, Sandeep K. Ganji, Joseph R. Goldenberg, Sabra C. Lewsey, Allison G. Hays, Robert G. Weiss, Michael Schär

**Affiliations:** ^1^ Division of Magnetic Resonance Research, Russell H. Morgan Department of Radiology and Radiological Science Johns Hopkins University School of Medicine Baltimore Maryland USA; ^2^ North America Clinical Science, MR R&D, Philips Cambridge Massachusetts USA; ^3^ Department of Radiology Mayo Clinic College of Medicine Rochester Minnesota USA; ^4^ Division of Cardiology Johns Hopkins University School of Medicine Baltimore Maryland USA

## Abstract

**Purpose:**

To simultaneously measure skeletal muscle energetics and blood flow (BF) before, during, and after dynamic plantar flexion exercise (PFE).

**Methods:**

Non‐localized pulse‐acquire phosphorus‐31 magnetic resonance spectroscopy (^31^P MRS) and phase contrast flow magnetic resonance imaging (^1^H MRI) using golden‐angle rotated spiral readouts were acquired in an interleaved fashion. Data were collected at rest, during PFE to volitional fatigue, and post‐exercise recovery in 10 healthy adults. Interleaved popliteal artery BF was validated against conventional cine phase‐contrast measures at rest and post‐exercise recovery, while calf muscle energetics were validated by comparing to conventional ^31^P MRS‐only measures at rest. To minimize motion artifacts during dynamic PFE, flow data were resolved in 3 dimensions (cardiac phase, pedal position, and exercise stage).

**Results:**

Resting phosphocreatine (PCr) and inorganic phosphate concentrations were similar between both techniques (both *p* > 0.05). During PFE, PCr declined to 46% ± 10% of rest. PCr recovery time was 43 ± 15 s. Resting BF was 132 ± 43 mL/min and increased up to four‐fold during PFE depending on exercise stage and pedal position. Notably, there was excellent agreement between the interleaved and conventional BF measures (*r* ^2^ = 0.95, slope = 0.977, *p* < 0.0001) with minimal bias (12.62 mL/min) over a wide range of physiologically relevant BF values (from ∼60 to 600 mL/min).

**Conclusion:**

The feasibility of simultaneous assessment of skeletal muscle energetics and BF during dynamic exercise by interleaved ^31^P MRS/^1^H flow MRI was demonstrated along with good agreement between interleaved and conventional techniques during rest and recovery. One can now simultaneously quantify critical BF‐metabolism relationships and use this to probe disease‐related adaptations.

## 1 Introduction

Impaired skeletal muscle energetic response to dynamic exercise is an important marker of disease in a variety of populations, including those with age associated frailty, muscle myopathies, diabetes, peripheral artery disease, and heart failure [1, 2, 3, 4, 5, 6]. In vivo phosphorus‐31 magnetic resonance spectroscopy (^31^P MRS) is a non‐invasive technique that allows for the dynamic assessment of high‐energy phosphate metabolites in skeletal muscle at rest, during dynamic exercise, and post‐exercise recovery [7]. Delayed phosphocreatine (PCr) recovery post‐exercise is closely associated with reduced maximal mitochondrial oxidative phosphorylation [8, 9, 10, 11] and differentiates disease from control participants [1, 2, 3, 4, 12]. Recent work has shown that the rate of PCr decline during exercise can also differentiate between disease and control, and may be more closely related to exercise intolerance [1, 5, 12, 13, 14]. However, it is not known whether these observations are reflective of a primary metabolic abnormality, or whether they are a secondary consequence of impaired skeletal muscle blood flow and oxygen delivery to the mitochondria (or a combination of both). An impaired blood flow response to exercise could be a key contributing factor leading to metabolic abnormalities, and a blunted blood flow response to exercise has been reported in a variety of populations, including heart failure [15, 16], peripheral artery disease [17], cancer survivors [18], and diabetes [19]. However, to date, methodological limitations have not allowed for the simultaneous assessment of muscle energetics and blood flow during dynamic exercise. Therefore, we aimed to develop an MR‐based method that interleaves ^31^P MRS with phase‐contrast proton (^1^H) MRI to achieve simultaneous metabolic and blood flow assessment before, during, and after dynamic exercise.

Interleaved ^31^P MRS and ^1^H MRI has been used as early as 1981 by Thulborn et al. [20] to study oxygen utilization and high‐energy phosphate metabolism in rabbit skeletal muscle. More recent studies have interleaved ^31^P MRS and ^1^H MRI to simultaneously assess metabolism and oxygen delivery/utilization in humans [21, 22, 23, 24, 25]. Most approaches for measuring arterial blood flow or muscle perfusion, whether interleaved with ^31^P MRS [21, 22, 23, 24, 25] or assessed in isolation [26, 27, 28, 29, 30, 31, 32, 33, 34, 35], have been limited to post‐exercise recovery [23, 24, 25, 26, 27, 28, 29, 33] or to changes in response to limb ischemia induced by cuff occlusion [23, 24, 30, 31, 32, 33, 34]. The main challenge is that motion artifacts during dynamic exercise compromise image quality and therefore make accurate blood flow assessment difficult.

One approach to avoid these limitations is to briefly pause exercise to acquire imaging data [22, 35]. However, arterial spin labeling perfusion imaging requires two acquisitions (tag/control), and exercise‐induced small spatial shifts lead to noisy perfusion signal during exercise compared to pre‐ and post‐exercise measures, making interpretation difficult. To overcome these methodological limitations, we developed a pulse sequence that measures blood flow during exercise with a phase contrast approach [36] using golden‐angle (GA) rotated variable density spiral readouts repeated in perpetuity and interleaved every 2 s with a pulse‐acquire non‐localized ^31^P MRS [37]. The GA flow data are then binned along different dimensions (cardiac phase, exercise stage, pedal position) and reconstructed with a sparse reconstruction to compensate for the exercise‐induced motion artifacts. To validate this technique, we compared high‐energy phosphate concentrations measured at rest and popliteal artery blood flow measured both at rest and during recovery using the new interleaved protocol to those of conventional ^31^P MRS and cine phase‐contrast MRI acquisitions acquired in isolation.

## 2 Methods

### 2.1 Study Participants

All human studies were approved by the Johns Hopkins School of Medicine Institutional Review Board and written informed consent was obtained from all study participants. Ten participants (five female, 34.3 ± 12.8 years, BMI 26.35 ± 4.60 kg/m^2^) with no history of hypertension, diabetes mellitus, or cardiovascular disease were enrolled.

### 2.2 Data Acquisition Protocol

**FIGURE 1:**
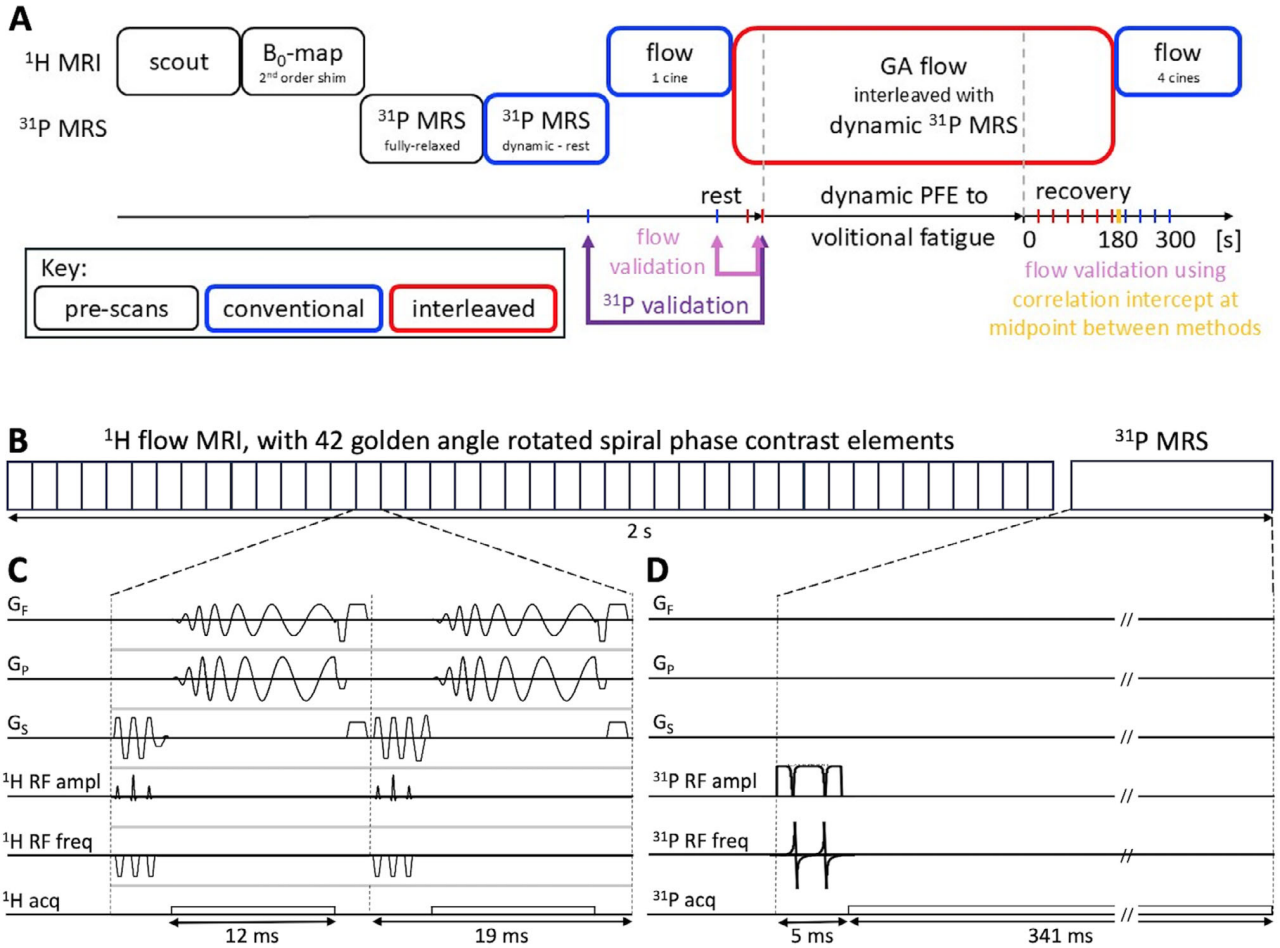
(A) Data acquisition protocol starting with ^1^H MRI scout and B_0_‐map for second order shimming, and fully‐relaxed ^31^P MRS to determine resting metabolite concentrations. The interleaved ^31^P MRS/^1^H flow MRI (shown in red) was validated against conventional (shown in blue) ^31^P‐MRS at rest, and cine flow acquisitions both at rest and during recovery. Recovery BF for each technique was defined as the regression intercept at the midpoint between the last interleaved acquisition and the first conventional acquisition (at 195 s of recovery shown in yellow). (B) Schematic of the interleaved ^31^P MRS/^1^H flow MRI pulse sequence alternating between 42 ^1^H MRI flow encoded acquisitions with golden angle rotated spiral readouts and a non‐localized pulse‐acquire ^31^P MRS acquisition. (C) Each of the 42 flow MRI elements contained two velocity encoded phase contrast acquisitions with a TR of 19 ms, water selective spectral‐spatial excitation, and variable density spiral readouts (12 ms). The spiral trajectory was rotated by the golden angle from one flow element to the next. (D) The non‐localized ^31^P MRS acquisition used a BIR‐4 adiabatic excitation pulse with a 341‐ms readout acquired every 2 s. acq, acquisition window; ampl, amplitude; freq, frequency; G_F_, frequency encoding gradient; G_P_, phase encoding gradient; G_S_, slice encoding gradient.

Participants were semi‐supine on the scanner table with a minimal bend in the knee and the foot of their dominant leg against an MRI‐compatible plantar flexion exercise device (Figure S1), with the ankle and knee of the exercising leg strapped to minimize contributions from other muscles during plantar flexion exercise, as previously reported [1, 5, 14]. Vector ECG was used for cardiac synchronization [38]. ^31^P MRS/^1^H flow MRI data were collected in a 3T MR system (Achieva, Philips Healthcare) using a 14‐cm diameter ^31^P‐transmit and receive single loop coil (Philips Healthcare) centered under the widest portion of the calf, and a two‐channel 15 cm diameter ^1^H Flex M coil located on the medial and lateral sides of the knee of the exercising leg. The data acquisition protocol (Figure 1A) was: (1) scout images to confirm optimal coil positioning; (2) static field B_0_‐map for localized second order shimming [39]; (3) fully relaxed non‐localized ^31^P MRS with TR = 25 s; (4) conventional dynamic ^31^P MRS at rest; (5) conventional cine phase contrast ^1^H MRI at rest; (6) interleaved ^31^P MRS/^1^H flow MRI at rest (2 min), during exercise (until volitional exhaustion), and through 180 s of recovery; (7) followed immediately by four conventional cine phase contrast ^1^H MRI during recovery. The exercise protocol has been detailed elsewhere [1, 5, 14]. Briefly, plantar flexion exercise was performed at a 1 Hz rate guided by a metronome, with a starting resistance of 0.9 kg which increased to 1.8 kg after 120 s and increased by 1.8 kg every 120 s thereafter until volitional fatigue. The interleaved ^31^P MRS/^1^H flow MRI pulse sequence (Figure 1B–D) consisted of an alternate collection of non‐localized pulse‐acquire ^31^P MRS spectra (TR = 2 s; flip angle = 50° using adiabatic BIR‐4 pulses; acquisition delay = 0.2 ms; bandwidth = 3000 Hz; 1024 spectral points) and 42 GA rotated ^1^H MRI spiral readouts with two phase contrast velocity encodes for each spiral interleave (TR = 19 ms; TE = 2.8 ms; readout duration = 12 ms, variable density spirals [center 30% of k‐space is fully sampled with 11 interleaves and outer 40% is under‐sampled by a factor of two with a linear transition in between], GA = 137.508°, water selective excitation with a spectral‐spatial 1‐2‐1 pulse, flip angle = 20°; FOV = 150 × 150 × 8 mm^3^; voxel size = 0.8 × 0.8 × 8 mm^3^, maximum encoded velocity (*v*_enc_) was set to ±200 cm/s). The conventional ^31^P MRS and ^1^H flow MRI sequences had the same parameters as used in the interleaved sequence, except that the conventional, vendor provided cine phase contrast sequence used retrospective ECG‐gating to 40 cardiac phases, 16 spiral interleaves to fully sample each image frame, velocity encodes were alternated every heartbeat leading to a 32‐heartbeat acquisition duration, and the *v*_enc_ for the rest acquisition was ±100 cm/s.

### 2.3 Image Reconstruction

Phase contrast data were reconstructed offline using the graphical programming interface (GPI) [40]. Golden angle spiral data were first binned along two dimensions during rest and recovery or three dimensions during plantar flexion exercise: (1) cardiac phases based on the ECG triggers, (2) rest, exercise, or recovery stages, and (3) during plantar flexion exercise only, four different foot pedal positions based on a pressure transducer placed under the foot pedal. Images were then reconstructed using k‐t sparse parallel imaging reconstruction adapted for phase contrast [41] using total variation sparsity constraints in up to four dimensions (cardiac, stages, pedal position, and flow encodes). 40 cardiac phases were applied during rest and recovery, but only 15 phases during exercise because data were split into different foot pedal positions as well. During rest and exercise, cine phase contrast data were created every minute and data from the final minute of each stage were analyzed to best reflect a physiological steady state. For participants that reached the point of volitional fatigue in less than 30 s into a new exercise stage, data were insufficient to reconstruct phase contrast images for that stage, and those data were included in the reconstruction for the previous exercise stage to ensure that the last flow images contained data from peak exercise. During recovery, cine phase contrast data were created every 30 s, leading to six recovery time points, and each was analyzed for assessment of popliteal blood flow.

### 2.4 Data Analysis

Phase‐contrast images (both conventional and interleaved) were first deblurred in GPI [41] and then analyzed using a custom MATLAB script (version R2020a, Mathworks, Natwick, MA, USA) [1]. A circular region of interest (ROI) was drawn around the popliteal artery for each cardiac phase followed by a background ROI in the surrounding tissue which was used to subtract the background phase. Care was taken to avoid veins and areas of high fat content in the background ROI. The popliteal ROI was used to determine cross‐sectional area and mean velocity in each cardiac phase. Popliteal artery blood flow was calculated as the product of the mean cross‐sectional area and the absolute of the mean velocity.

The ^31^P spectra were first apodized using a 5 Hz Lorentzian filter in the time domain, and then zero and first‐order phase corrected in the frequency domain. ^31^P MRS spectra (fully relaxed and both dynamic conventional and interleaved) were fitted using the AMARES function in jMRUI [42]. From the fully relaxed data, the PCr/γ‐ATP was determined, and absolute PCr concentration was determined from this ratio and assuming a resting ATP concentration of 5.58 μmol/g wet weight for each participant [7, 43]. The PCr SNR was calculated by dividing the amplitude of the PCr peak by the standard deviation of the noise measured from the signal‐free areas in the time domain [44]. Spectra from the last 20 s of each stage, including rest, each subsequent exercise stage, and the last 20 s prior to when self‐reported fatigue was reached and exercise halted, were averaged and then fit using the same AMARES fitting tool in jMRUI [42]. Intracellular pH was calculated from the chemical shift differences between the PCr and inorganic phosphate resonances, using a weighted sum of the two inorganic phosphate peaks when they split during exercise [45, 46, 47]. Changes in PCr, ATP, and inorganic phosphate concentrations from rest to exercise were determined based on these averaged data. The rate of PCr decline was calculated as the decrease in PCr from rest to the point of fatigue normalized for the work performed, as previously described [1, 5, 14]. To determine the PCr recovery time, the serial single‐average post‐exercise PCr data were then fit to a mono‐exponential function in a custom‐built MATLAB script [1, 48, 49].

### 2.5 Statistical Analysis

Differences between the conventional and interleaved approaches were tested with paired *t*‐tests and reported as mean ± SD when normally distributed or Wilcoxon rank sum tests and reported as median (interquartile range) when not normally distributed. Methodological bias was determined by Bland–Altman plots. Changes in exercise blood flow from rest to peak exercise and from the first plantar flexion exercise stage to the last were compared by paired *t*‐tests. For the recovery blood flow comparisons, regression slope and intercept of the recovery blood flow response were determined from either the six recovery time points of the interleaved method or the four conventional cine phase contrast method. The regression intercept was determined at the midpoint between the end of the final interleaved recovery acquisition and the first conventional cine acquisition (at 195 s of recovery). Slope and intercept were compared between the two methods by paired *t*‐tests or Wilcoxon sum rank tests. All statistical analyses were performed in GraphPad Prism (version 10.4.1 for Windows, GraphPad Software, Boston, MA, USA) with *α* = 0.05.

## 3 Results

Popliteal artery blood flow and skeletal muscle energetic measures were successfully acquired in all 10 participants at rest, during dynamic exercise, and post‐exercise recovery using the interleaved protocol.

### 3.1 High‐Energy Phosphate Metabolism

**FIGURE 2:**
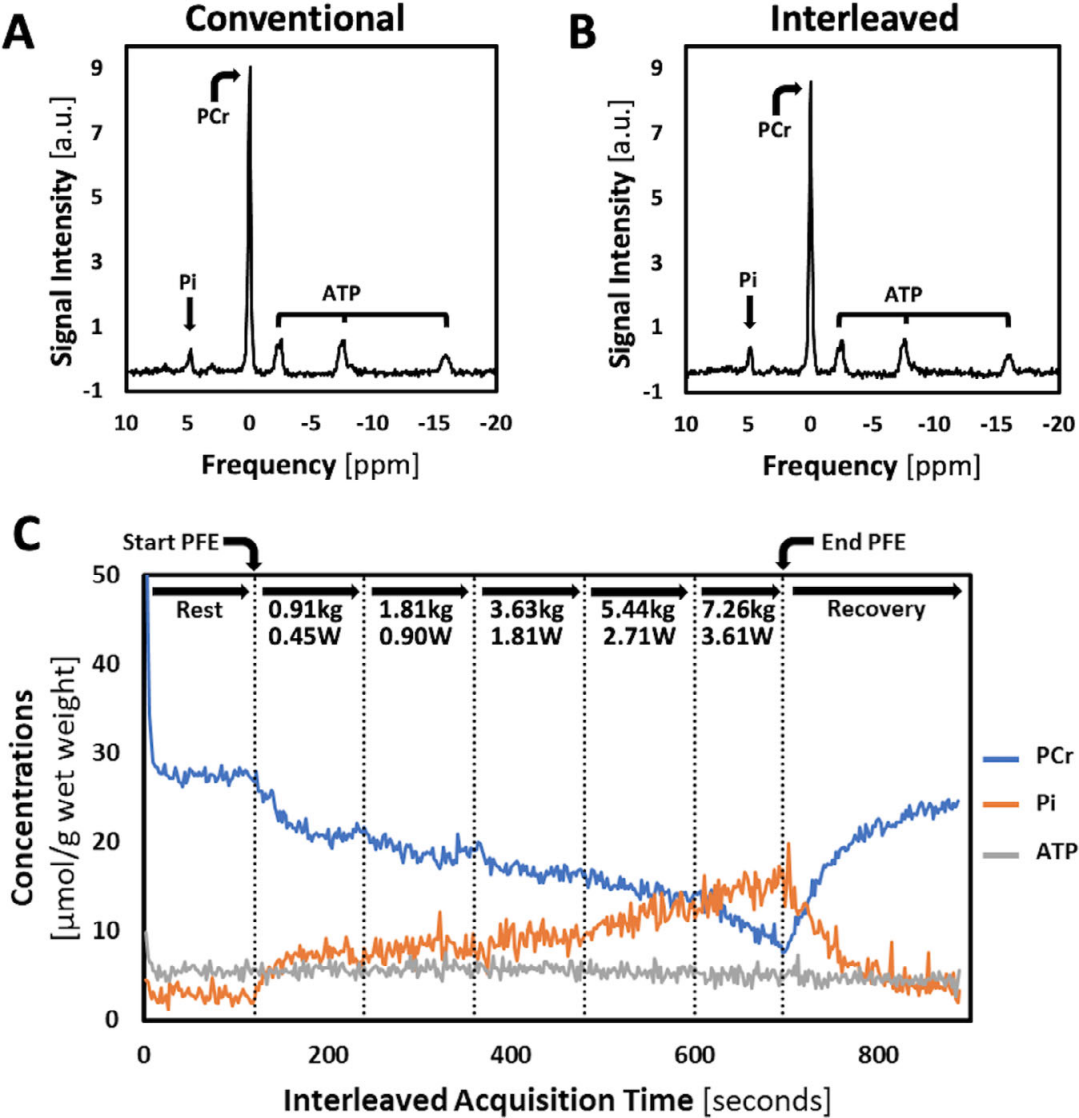
Representative ^31^P spectra acquired using either the conventional (A) or interleaved ^31^P MRS/^1^H MRI approach (B) in the same individual (1 average; TR = 2 s). The time course of phosphocreatine (PCr), inorganic phosphate (Pi), and adenosine triphosphate (ATP) dynamics acquired with the interleaved approach are shown in the same participant (C). As expected, PCr gradually declines during plantar flexion exercise (PFE) with a concomitant increase in Pi, while ATP is maintained throughout. The resistance in kg and power output in Watts are reported for each exercise stage.

**FIGURE 3:**
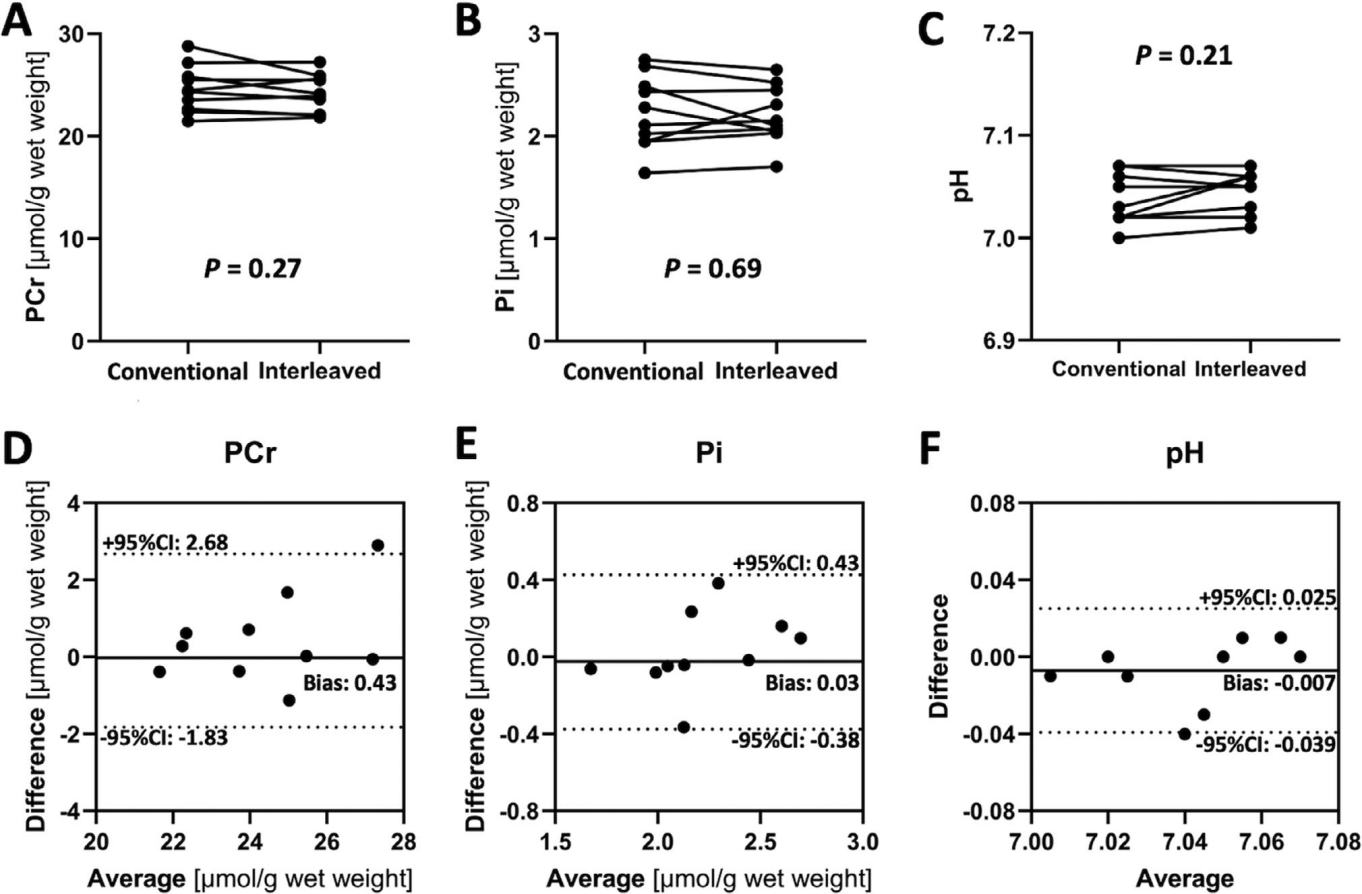
Resting phosphocreatine concentration (PCr; A), inorganic phosphate concentration (Pi; B), and intracellular pH (C) were similar between those measured using the interleaved and conventional sequences in all subjects. Bland–Altman plots support this by demonstrating minimal bias in measurements of PCr (D), Pi (E), and pH (F).

To validate the ^31^P MRS measures acquired with the interleaved approach, the metabolite measures were compared to those of a conventional ^31^P MRS acquisition performed prior to the interleaved acquisition at rest when metabolite pool sizes were not changing between interleaved and conventional acquisitions. Example resting spectra are shown in Figure 2A,B. Resting PCr and inorganic phosphate metabolite concentrations as well as resting intracellular pH were similar between those measured using the interleaved and the conventional MRS pulse sequences with minimal bias (all *p* > 0.05; Figure 3). Moreover, the PCr SNR were similarly high between both protocols (8.44 ± 1.48 vs. 8.40 ± 1.39, interleaved vs. conventional, respectively; *p* = 0.87).

During plantar flexion exercise, all participants reached the third exercise stage, nine participants reached the fourth exercise stage, four participants reached the fifth exercise stage, and one participant reached the sixth exercise stage. During the exercise portion of the interleaved protocol, we observed a decline in PCr to a mean 46% ± 10% of resting values with a concomitant increase in inorganic phosphate concentrations to 592% ± 121% of resting values. ATP concentrations did not change from rest to fatigue (97% ± 8% of rest). Intracellular pH decreased from 7.04 ± 0.02 at rest to 6.89 ± 0.16 at fatigue (*p* = 0.0067). The average PCr decline during exercise was 13.1 ± 18.2 μmol/g wet weight/kJ and the PCr recovery time post‐exercise measured using the interleaved approach was 46 ± 17 s.

### 3.2 Phase Contrast Popliteal Artery Blood Flow

**FIGURE 4:**
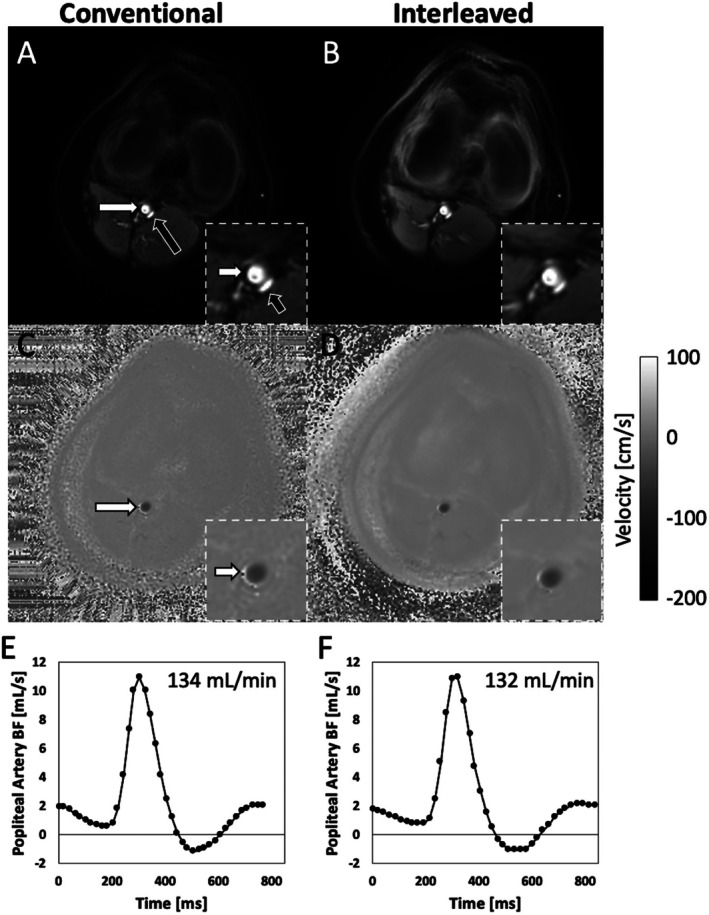
Phase‐contrast magnitude (A and B) and velocity (C and D) images from the same participant as Figure 2 at rest prior to exercise acquired using the conventional cine approach (A and C) and the interleaved approach (B and D). The images shown are from the 16th of 40 cardiac phases where peak popliteal artery blood flow (BF) is observed. The insets show a zoomed area containing the popliteal artery. The white and black arrows identify the popliteal artery and vein respectively. Negative velocities, shown as darker image intensity, indicate flow in head‐foot direction. The popliteal artery flow profile throughout the cardiac cycle was similar between the two methods (E and F). The mean BF is listed in the top right of panels E and F.

To validate the blood flow measures acquired with the interleaved approach, conventional cine phase contrast MRI was performed both at rest prior to the interleaved acquisition and during recovery from exercise immediately after the interleaved acquisition. Phase‐contrast magnitude images and velocity maps acquired at the level of the knee prior to exercise using the interleaved protocol (Figure 4A–D) clearly shows the popliteal artery and vein. Visually, the image quality from the interleaved approach is similar to that acquired using the conventional cine phase contrast only approach (Figure 4A–D, Video S1). As a result, the popliteal artery blood flow profile was similar between the two methods (Figure 4E,F).

**FIGURE 5:**
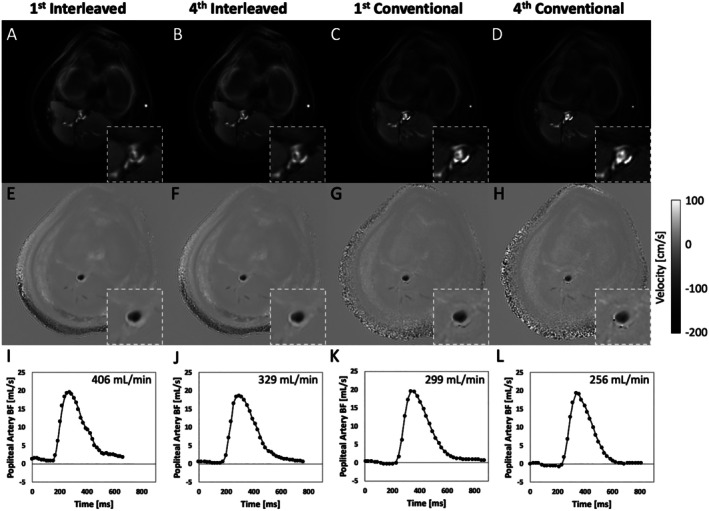
In the same representative participant as Figures 2 and 4, phase‐contrast magnitude (A–D) and velocity (E–H) images acquired during recovery from exercise with the interleaved approach (A, B, E, F) and the conventional cine approach (C, D, G, H) are shown. The images shown are from the 16th of 40 cardiac phases around where peak popliteal artery blood flow (BF) is observed. Shown are the first and fourth of six interleaved flow measures acquired 30 and 120 s after exercise cessation, and the first and fourth of four conventional cine flow measures acquired 210 and 300 s after exercise. The insets show a zoomed area containing the popliteal artery. Negative velocities, shown as darker image intensity, indicate flow in head‐foot direction. The popliteal artery flow profiles throughout the cardiac cycle for each measurement (I–L) show a gradual decrease of the recovery flow back towards resting values. The mean BF results for each recovery measure are listed in the top right of panels I–L.

**FIGURE 6:**
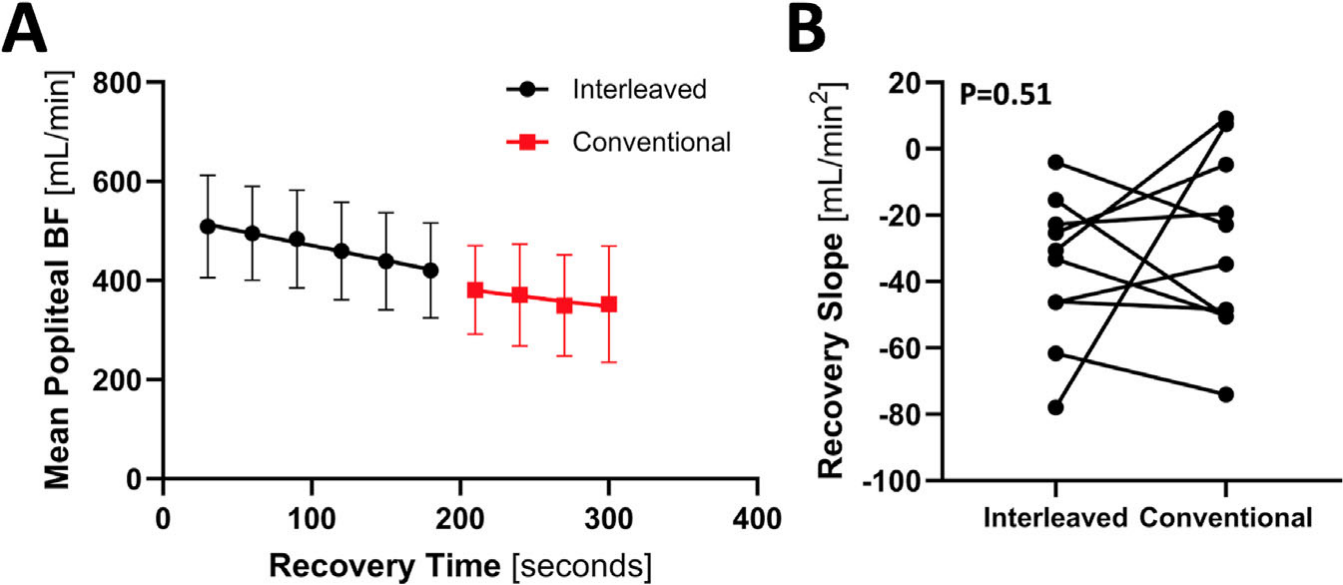
The grouped change in mean popliteal blood flow (BF) during recovery is shown in (A), with the first six measures acquired with the interleaved approach (black circles and lines) and the last four measures acquired with the conventional cine approach (red squares and lines). Data are shown as grouped mean and standard deviation. There was no significant difference in the regression fit slope between the two approaches (B).

**FIGURE 7:**
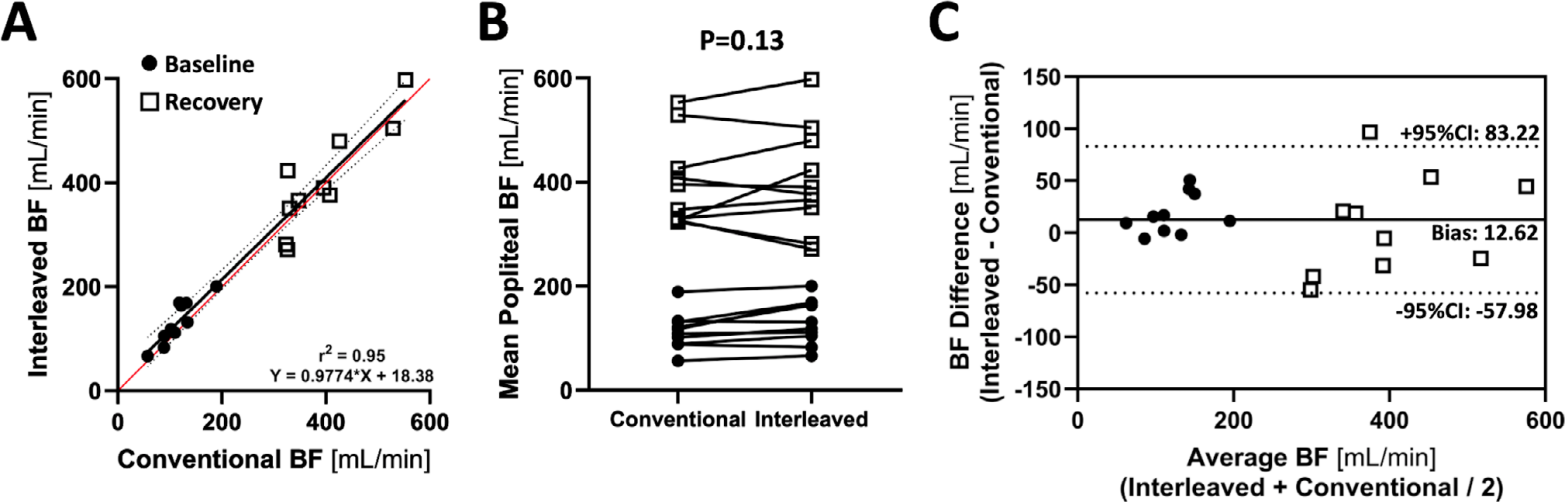
Popliteal artery blood flow (BF) was assessed using both the interleaved and conventional approaches at rest (closed circles) and during recovery (open squares) from plantar flexion exercise to test the validity of the interleaved approach across a wide physiological range of BF. Shown are (A) a strong positive correlation with a slope of 0.98, (B) a lack of significant differences in paired comparisons, and (C) small bias as shown in the Bland–Altman plots. The red line in (A) represents the line of identity, and the regression equation and *r* ^2^ are stated on the figure. CI, confidence interval.

During post‐exercise recovery, the phase‐contrast images and flow profiles maintained similarly high visual quality as those acquired at rest for both the interleaved and the conventional approach (Figure 5A–F, Video S2). During recovery, the regression slope of the popliteal artery blood flow did not change when switching from the interleaved sequence to the conventional sequence (slope: −36.3 ± 22.1 mL/min^2^ vs. −28.9 ± 27.6 mL/min^2^, *p* = 0.51; Figure 6). When comparing the blood flow data acquired using the interleaved and conventional approaches across a wide physiological range (both rest and post‐exercise recovery), there was excellent agreement between the two techniques (*r* ^2^ = 0.95, slope = 0.977, *p* < 0.0001; Figure 7A). Moreover, there were no significant differences between the two techniques (*p* = 0.13; Figure 7B) and very little bias was observed (bias: 12.62 mL/min; 95% CI −57.98 to 83.22 mL/min; Figure 7C).

**FIGURE 8:**
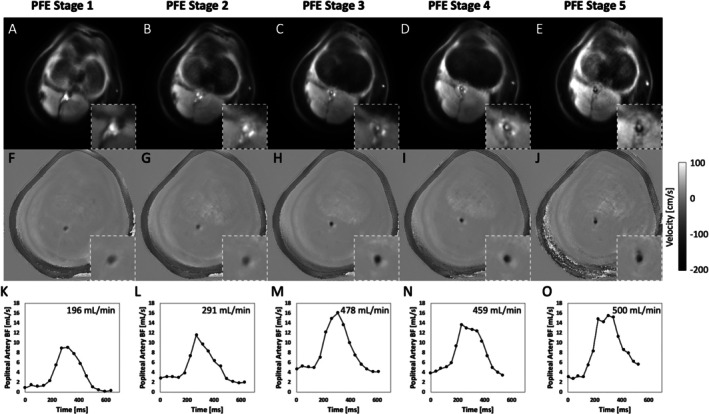
Phase contrast magnitude (A–E) and velocity (F–J) images acquired during plantar flexion exercise (PFE) with the interleaved approach in the same participant as in Figures 2, 4 and 5. Shown are the ninth of 15 cardiac phases demonstrating near‐peak popliteal artery blood flow (BF). Shown are the last (second) flow images from each PFE stage with the foot in the fully relaxed pedal position. The insets show a zoomed area of the popliteal artery. Negative velocities, shown as darker image intensity, indicate flow in head‐foot direction. The corresponding popliteal artery BF profiles throughout the cardiac cycle are shown below (K–O). The flow profile is almost entirely above zero during the first stage of PFE (K), and the baseline and peak of the BF profile shift significantly upwards as exercise intensity increases. The mean BF results for each PFE stage are listed in the top right of panels K–O.

**FIGURE 9:**
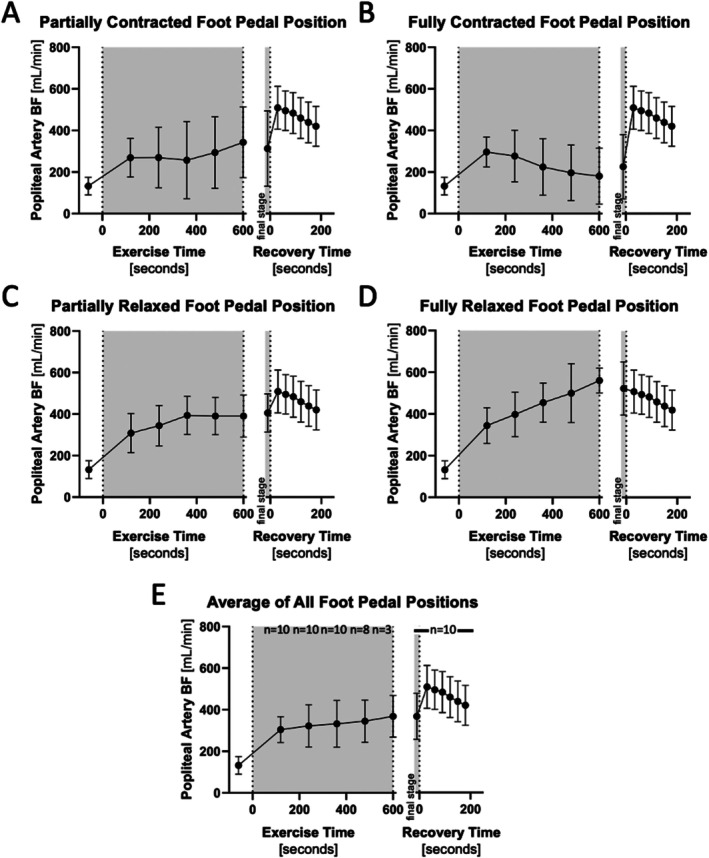
The grouped change in popliteal artery blood flow (BF) during rest, dynamic PFE (shaded gray), and recovery is shown for each of the four PFE foot pedal positions: Partially contracted (A), fully contracted (B), partially relaxed (C), and fully relaxed (D). The flow response during the final stage of PFE was lower when the calf muscles were fully contracted to cause flexion around the ankle joint (B), as compared to when the foot was in fully relaxed position to cause dorsi flexion around the ankle joint (D), and intermediate for the two positions in between (A and C). As a result, popliteal BF overshoots from the end of PFE to the beginning of recovery once all four foot pedal positions are averaged (E). Plots represent mean ± SD. The sample size at each exercise stage is noted in panel E but are consistent across all other panels (A–D). The decrease in sample size with greater exercise intensity reflects the drop out in participants due to reaching fatigue. The average BF in the final PFE stage is plotted before the recovery curves.

During dynamic plantar flexion exercise, popliteal artery blood flow data was available for all participants for three exercise stages, eight participants for four exercise stages, and three participants for five exercise stages. The leg moved during each pedal stroke and also as the exercise resistance increased over time. Binning the data into four pedal positions and one‐minute exercise stages enabled to sufficiently suppress motion artifacts. Example phase contrast images and blood flow profiles acquired during plantar flexion exercise with the foot pedal in the relaxed position are shown in Figure 8. Video S3 shows phase contrast magnitude and velocity cines for all stages and across all four pedal positions in the same participant. To better demonstrate the motion occurring during plantar flexion exercise, Video S4 shows a single cardiac phase (phase 9 of 15) animated along pedal positions and exercise stages. Figure 9 shows the popliteal artery blood flow measured using the interleaved approach for each of the four foot pedal positions (A‐D) and the average of all pedal positions (E). Blood flow increased from 132 ± 43 mL/min at rest to up to 345 ± 85 mL/min (*p* < 0.0001) at the end of the first exercise stage, and up to 524 ± 128 mL/min (*p* < 0.0001) at peak exercise. This ∼4‐fold blood flow increase was observed when the foot pedal was in the fully relaxed position (Figure 9D) and its flow response was higher compared to the other pedal positions (Figure 9A–C) where muscle contraction is present. Although there was no significant difference in blood flow measured at different pedal positions during the first exercise stage, we observed lower blood flow at peak exercise when the pedal was in partially (*p* = 0.0077) or fully contracted positions (*p* = 0.0005) compared to the fully relaxed position.

Comparing blood flow at peak exercise with the first measure during recovery, there was no change (1% ± 24%, *p* = 0.64) when looking at the exercise flow from the fully relaxed foot pedal position (Figure 9D). However, if we consider the average of all foot pedal positions during exercise, including those where muscle contraction takes place, then there was an apparent 49% ± 45% overshoot (*p* = 0.0051; Figure 9E) in popliteal artery blood flow observed immediately post exercise cessation. Figure S2 shows popliteal artery blood flow from each foot pedal position in all subjects at all stages acquired with the interleaved approach and the conventional cine approach.

## 4 Discussion

We developed and implemented an interleaved ^31^P MRS/^1^H flow MRI approach that enables simultaneous acquisitions of non‐localized pulse‐acquire ^31^P MR spectroscopy and phase contrast velocity‐sensitive ^1^H MRI using GA rotated variable density spiral readouts in an interleaved fashion. The interleaved approach was successfully applied in 10 participants at rest, during dynamic plantar flexion exercise, and post exercise during recovery. Measured metabolite concentrations were validated against conventional ^31^P MRS at rest, and blood flow measures were validated against conventional cine phase‐contrast ^1^H MRI acquisitions over a wide range of physiologically relevant blood flow values acquired during rest and post‐exercise recovery. The feasibility of simultaneous metabolites and blood flow measures during dynamic plantar flexion exercise was demonstrated.

The interleaved ^31^P MRS/^1^H flow MRI was first validated at rest with similar PCr SNR and resting PCr and Pi concentrations measured by the interleaved method and the conventional ^31^P MRS‐only approach. Some previous work interleaving ^31^P MRS/^1^H MRI, where both acquisitions imaged the same tissue, has shown metabolite specific signal and SNR increase through nuclear Overhauser enhancement [22, 23, 50, 51]. Here, we observed no enhancement because the ^1^H flow MRI slice orthogonal to the popliteal artery is placed at the level of the knee without overlapping the muscle tissue observed by the ^31^P coil. During dynamic exercise, detected metabolite changes were similar to our previous work [14] (shown in brackets), PCr decreased to 46% ± 10% (41% ± 16%) of resting values, and Pi increased to 592% ± 121% (531% ± 159%) of rest, in order to maintain stable ATP concentrations, which were 97% ± 8% (97% ± 8%) of rest. Despite only acquiring an abbreviated period of post‐exercise recovery ^31^P MRS data, the PCr recovery time measured herein (43 ± 15 s) is similar to those reported previously by our group (46 ± 17 s [14]) and in line with the expert consensus paper (41 ± 3 s [7]).

Resting popliteal artery blood flow was on average 132 ± 43 mL/min measured by the interleaved approach which is similar to previous reports in healthy controls by velocity encoded MRI (∼80–100 mL/min [33]) and vascular ultrasound (140 ± 54 mL/min and 110 ± 43 [52, 53]). Because blood flow gradually returns towards resting values during post‐exercise recovery, we compared the regression slope of the interleaved and conventional acquisitions and found no differences between the two techniques. When combining the popliteal artery blood flow measures at rest and recovery, there was excellent agreement between the measurements from the interleaved and the conventional techniques across a range of blood flow values from 60 to 600 mL/min. No significant group differences in blood flow measurements between both techniques were observed; they were strongly correlated, Bland–Altman plots indicated minimal bias, and the regression slope of almost 1 combined with narrow confidence intervals and low mean absolute error demonstrated the precision and accuracy of the interleaved technique.

Blood flow measures during dynamic exercise have previously been difficult, particularly using MR‐based methods. Artifacts caused by motion between label and control acquisitions prevent accurate muscle perfusion measures during dynamic exercise using arterial spin labeling approaches. The interleaved method aims to avoid these issues by continuously acquiring GA‐rotated spiral phase contrast data throughout the acquisition period which are then binned according to stage of exercise, cardiac phase, and foot pedal position during image reconstruction. Binning the data according to the foot pedal position reduces the impact of motion on image quality, resulting in successfully capturing a near linear increase in popliteal artery blood flow from rest to peak exercise in the relaxed foot pedal position. This demonstrates the feasibility of blood flow measures during dynamic exercise using the interleaved approach, which has the potential to provide important insights into disease adaptations that are only unmasked during acute exercise. This advancement overcomes previous methodological limitations, potentially allowing for a more nuanced understanding of how different diseases affect peripheral vascular function and their interactions with metabolism. Using this technique, on average we observed a ∼4‐fold increase in popliteal artery blood flow from rest to peak exercise when the foot was in a fully relaxed pedal position (Figure 9D). This is a similar increase from rest to immediately post exercise in MRI‐based measures of popliteal vein blood flow [33]. The almost linear blood flow response to increasing exercise intensity is similar to what has been seen in the femoral artery measured by either Doppler echocardiography [54, 55, 56, 57] or via invasive thermodilution [58, 59]. When the foot was in a fully contracted position, however, we observed an attenuated increase in popliteal artery blood flow from rest to the final stage of exercise. The linear decrease in blood flow (Figure 9B) with increasing load suggests that intermuscular pressure increased to perform the increasing mechanical work and possibly compressed blood flow [60, 61].

The main limitation of this study was a lack of a simultaneous gold‐standard measure during dynamic exercise to directly validate the interleaved technique. Participants could theoretically complete three bouts of exercise, one during the interleaved acquisition and one with each conventional protocol. However, inconsistencies in effort, physical ability, warm‐up effects, and inherent physiological variation with different bouts of exercise in a given individual can impact the comparisons, independent of the acquisition protocol per se. Despite this limitation, the ability of the interleaved technique to detect physiologically relevant changes in metabolites and blood flow from rest to exercise in line with previously published data supports its validity. Moreover, the interleaved method measures macrovascular blood flow, in this case in the popliteal artery, and does not provide insight into microvascular blood flow or tissue perfusion, as is acquired using arterial spin labeling techniques. Yet, popliteal artery flow offers a measure of the blood flow to the entire lower leg, and not just a reflection of a single mid‐calf slice, which we consider a strength. Another limitation of the current setup is that in some of the participants the quality of the ECG signal degraded during exercise and required manual corrections of the detected cardiac triggers. In two subjects, we were unable to create triggers based on the ECG signal, and instead generated cardiac triggers based on the flow curve after a real‐time reconstruction.

While not a limitation to this steady‐state exercise modality, a temporal resolution of one flow profile every 1 min would not be suited to a shorter exercise stage and might underestimate peak exercise blood flow. Real‐time flow measurements with either complex difference MRI [62] or 2D spatial selective excitation [63], which have been demonstrated during and after cuff occlusion, might be alternative methods to improve the temporal resolution, but remain to be demonstrated during dynamic exercise. An additional limitation is that the localization of the ^31^P MRS signal is solely based on the receive profile of the ^31^P coil, where the main contribution will be from the activated gastrocnemius, but contamination from the less activated soleus [64] may lead to underestimations in the observed metabolic changes. Adding a localization scheme could alleviate this limitation [65].

## 5 Conclusion

The interleaved ^31^P MRS/^1^H flow MRI approach for the simultaneous assessment of skeletal muscle energetics and popliteal artery blood flow has been validated at rest and during post‐exercise recovery, and feasibility during dynamic exercise has been demonstrated. By interleaving pulse‐acquire non‐localized ^31^P spectra with phase contrast velocity selective ^1^H MRI using GA rotated variable density spiral readouts, and binning the data along three dimensions, it is possible to detect physiologically relevant high‐energy metabolite concentrations and popliteal blood flow changes at rest, during dynamic exercise, and post‐exercise recovery. This method has the possibility of providing new insights into the contributing mechanisms leading to impaired oxygen and other nutrient delivery and muscle metabolism and their impact on exercise intolerance and quality of life in a wide range of different clinical and sub‐clinical populations. Future work should seek to confirm the clinical utility of this technique in cross‐sectional and longitudinal studies.

## Funding

The primary funding for this investigation was from the National Institutes of Health (NIH) R01 HL61912, R01 AG095963, AG063661 and AG063661‐03S1, as well as K99 HL171873. Dr. Robert G. Weiss also has support from the Clarence Doodeman Endowment in Cardiology at Johns Hopkins. Dr. Sabra C. Lewsey is supported by Kirschstein‐NRSA T32HL007227, and the Johns Hopkins Older Americans Independence Center Junior Faculty Investigator Award from the Johns Hopkins University Claude D. Pepper Older Americans Independence Center P30AG021334. Dr. Allison G. Hays is supported by R35 HL172680.

## Conflicts of Interest

Sandeep K. Ganji is an employee of Philips, Cambridge, MA, United States.

## Supporting information


**Figure S1:** A photograph depicting the plantar flexion exercise device and its dimensions. The participants dominant foot was placed on the ∼30 cm tall foot pedal, which was attached to the resistance weights (drawn) via ropes and pulleys. The adjustable front and back stops on either side of the foot pedal were optimized for each participant so that each lifted the resistance weights by 5 cm with every plantar flexion. Exercise was performed at a 1 Hz rate guided by a metronome and monitored by a pressure bellow located on the back side of the foot pedal. The exercise rate, weight displacement, and resistance were standardized throughout the entire study, and participants were guided throughout the exercise test for compliance by a study team member.
**Figure S2:** The individual popliteal artery blood flow (BF) measured at rest, during dynamic PFE, and recovery is shown for each of the four PFE foot pedal positions, partially contracted (A), fully contracted (B), partially relaxed (C), fully relaxed (D), and the average of all four pedal positions (E). Data are shown for measurements made using the cine phase‐contrast (conventional) acquisition collected in isolation at rest and the end of recovery, and from the interleaved method throughout rest, dynamic exercise, and initial 3 min of recovery. While there is individual variation, the typical flow response during the final stage of PFE was lower when the calf muscles were fully contracted to cause flexion around the ankle joint (B), as compared to when the foot was in fully relaxed position to cause dorsi flexion around the ankle joint (D), and intermediate for the two positions in between (A and C). As a result, in most participants, popliteal BF increases from the end of PFE to the beginning of recovery once all four foot pedal positions are averaged (E).

**Video S1:** Phase contrast magnitude (top row) and velocity (bottom row) maps are shown as cine videos for the conventional approach (leftmost) and two consecutive 1 min resting stages acquired with the interleaved approach (right two images) in the same representative participant as in Figures 2, 4, 5, and 7. The image quality from the interleaved approach is similar to that acquired using the conventional cine phase contrast only approach. Negative velocities, shown as darker image intensity, indicate flow in head‐foot direction.

**Video S2:** In the same representative individual, phase contrast magnitude (top row) and velocity (bottom row) maps are shown as cine videos reconstructed every 30 s during post‐exercise recovery. The first six pairs of images are reconstructed from the interleaved acquisition followed immediately by four additional conventional phase‐contrast only acquisitions. The image quality is similar between the interleaved and the conventional approaches. Negative velocities, shown as darker image intensity, indicate flow in head‐foot direction.

**Video S3:** Phase contrast magnitude and velocity maps are shown as cine videos during dynamic plantar flexion exercise in the same representative individual. Images are from each PFE stage, beginning with the first PFE stage (leftmost) and finishing with the final PFE stage (rightmost). Each set of five magnitude and velocity maps are repeated below for each of the four foot pedal positions. The velocity maps demonstrate how the popliteal artery blood flow is impacted by the differing foot pedal positions. Negative velocities, shown as darker image intensity, indicate flow in head‐foot direction.

**Video S4:** The phase contrast magnitude (top) and velocity (bottom) map for the same representative participant is shown for a single cardiac phase (phase 9 of 15) animated along pedal positions and exercise stages. This demonstrates the motion occurring during exercise, highlighting the need to resolve foot pedal positions during image reconstruction of dynamic exercise to avoid motion artifacts and to accurately measure popliteal artery blood flow. Negative velocities, shown as darker image intensity, indicate flow in head‐foot direction.

## Data Availability

The data that support the findings of this study are available from the corresponding author upon reasonable request.
