# Supplementary material for: Simultaneous Assessment of Skeletal Muscle Energetics and Blood Flow During Dynamic Exercise by Interleaved 31P‐MRS/ 1H‐MRI
Source: Magn Reson Med. 2026 Mar 17;96(1):13–26. doi: 10.1002/mrm.70337 (PMC13077704; doi:10.1002/mrm.70337)
Supplement: Supplementary file 1 — Figure S1: A photograph depicting the plantar flexion exercise device and its dimensions. The participants dominant foot was placed on the ∼30 cm tall foot pedal, which was attached to the resistance weights (drawn) via ropes and pulleys. The adjustable front and back stops on either side of the foot pedal were optimized for each participant so that each lifted the resistance weights by 5 cm with every plantar flexion. Exercise was performed at a 1 Hz rate guided by a metronome and monitored by a pressure bellow located on the back side of the foot pedal. The exercise rate, weight displacement, and resistance were standardized throughout the entire study, and participants were guided throughout the exercise test for compliance by a study team member. Figure S2: The individual popliteal artery blood flow (BF) measured at rest, during dynamic PFE, and recovery is shown for each of the four PFE foot pedal positions, partially contracted (A), fully contracted (B), partially relaxed (C), fully relaxed (D), and the average of all four pedal positions (E). Data are shown for measurements made using the cine phase‐contrast (conventional) acquisition collected in isolation at rest and the end of recovery, and from the interleaved method throughout rest, dynamic exercise, and initial 3 min of recovery. While there is individual variation, the typical flow response during the final stage of PFE was lower when the calf muscles were fully contracted to cause flexion around the ankle joint (B), as compared to when the foot was in fully relaxed position to cause dorsi flexion around the ankle joint (D), and intermediate for the two positions in between (A and C). As a result, in most participants, popliteal BF increases from the end of PFE to the beginning of recovery once all four foot pedal positions are averaged (E). [file MRM-96-13-s004.pdf]

SUPPORTING INFORMATION

Simultaneous assessment of skeletal muscle energetics and blood flow during dynamic exercise by interleaved ^{31}P-MRS/^{1}H-MRI.

T. Jake Samuel,[1] Sandeep K. Ganji,[2,3] Joseph R. Goldenberg,[4] Sabra C. Lewsey,[4] Allison G. Hays,[4] Robert G. Weiss,[1,4] and Michael Schär[1]

[1]Division of Magnetic Resonance Research, Russell H. Morgan Department of Radiology and Radiological Science, Johns Hopkins University School of Medicine, Baltimore, MD, USA.

[2]North America Clinical Science, MR R&D, Philips, Cambridge, MA, USA.

[3]Department of Radiology, Mayo Clinic College of Medicine, Rochester, MN, USA.

[4]Division of Cardiology, Johns Hopkins University School of Medicine, Baltimore, MD, USA.

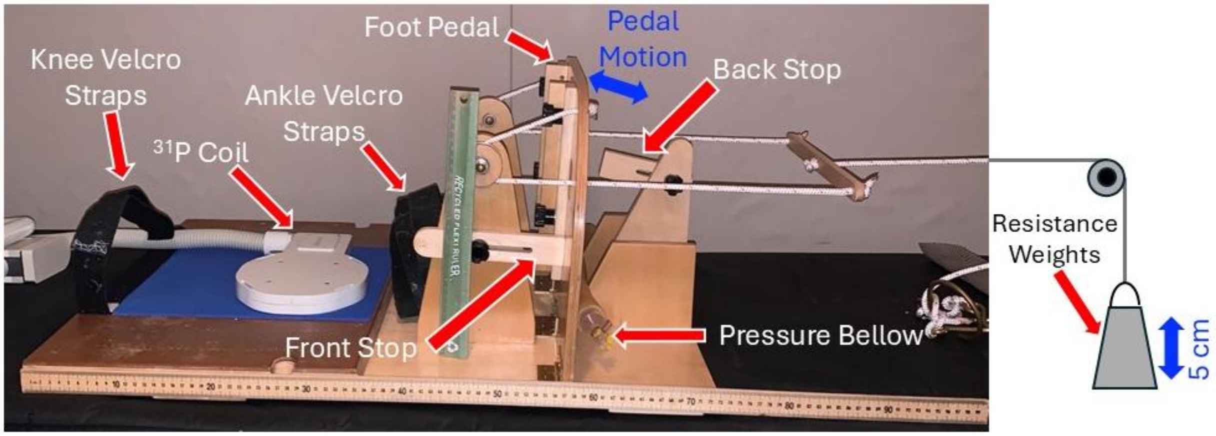

Supporting Information Figure S1: A photograph depicting the plantar flexion exercise device and its dimensions. The participants dominant foot was placed on the ~30cm tall foot pedal, which was attached to the resistance weights (drawn) via ropes and pulleys. The adjustable front and back stops on either side of the foot pedal were optimized for each participant so that each lifted the resistance weights by 5 cm with every plantar flexion. Exercise was performed at a 1 Hz rate guided by a metronome and monitored by a pressure bellow located on the back side of the foot pedal. The exercise rate, weight displacement, and resistance were standardized throughout the entire study, and participants were guided throughout the exercise test for compliance by a study team member.

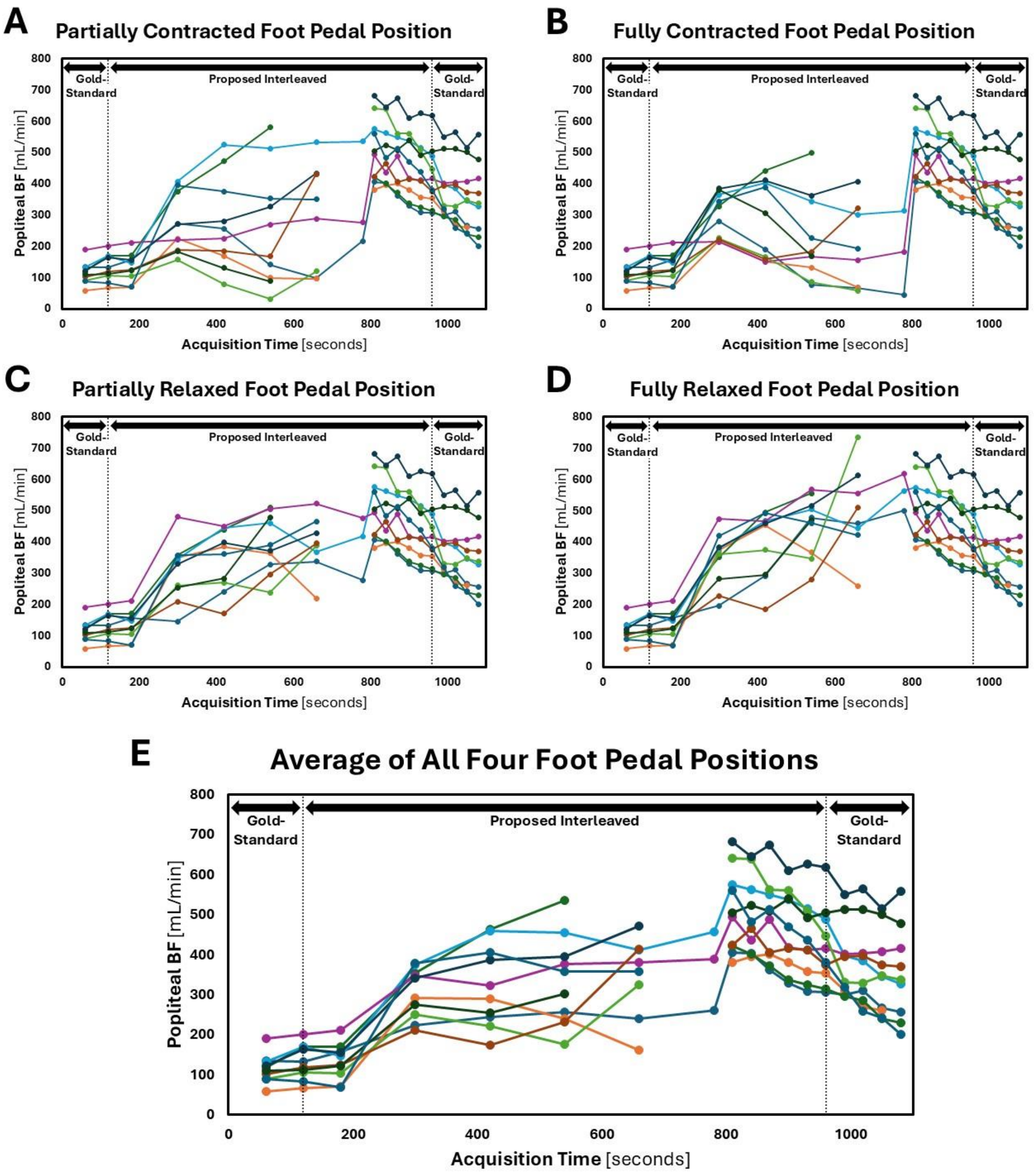

Supporting Information Figure S2. The individual popliteal artery blood flow (BF) measured at rest, during dynamic PFE, and recovery is shown for each of the four PFE foot pedal positions, partially contracted (**A**), fully contracted (**B**), partially relaxed (**C**), fully relaxed (**D**), and the average of all four pedal positions (**E**). Data are shown for measurements made using the cine phase-contrast (gold-standard) acquisition collected in isolation at rest and the end of recovery, and from the proposed interleaved method throughout rest, dynamic exercise, and initial 3 minutes of recovery. While there is individual variation, the typical flow response during the final stage of PFE was lower when the calf muscles were fully contracted to cause flexion around the ankle joint (**B**), as compared to when the foot was in fully relaxed position to cause dorsi flexion around the ankle joint (**D**), and intermediate for the two positions in between (**A,C**). As a result, in most participants, popliteal BF increases from the end of PFE to the beginning of recovery once all four foot pedal positions are averaged (**E**).
